# The Effect of Map Boundary on Estimates of Landscape Resistance to Animal Movement

**DOI:** 10.1371/journal.pone.0011785

**Published:** 2010-07-26

**Authors:** Erin L. Koen, Colin J. Garroway, Paul J. Wilson, Jeff Bowman

**Affiliations:** 1 Environmental and Life Sciences, Trent University, Peterborough, Canada; 2 Biology Department, Trent University, Peterborough, Canada; 3 Wildlife Research and Development Section, Ontario Ministry of Natural Resources, Peterborough, Canada; University of Fribourg, Switzerland

## Abstract

**Background:**

Artificial boundaries on a map occur when the map extent does not cover the entire area of study; edges on the map do not exist on the ground. These artificial boundaries might bias the results of animal dispersal models by creating artificial barriers to movement for model organisms where there are no barriers for real organisms. Here, we characterize the effects of artificial boundaries on calculations of landscape resistance to movement using circuit theory. We then propose and test a solution to artificially inflated resistance values whereby we place a buffer around the artificial boundary as a substitute for the true, but unknown, habitat.

**Methodology/Principal Findings:**

We randomly assigned landscape resistance values to map cells in the buffer in proportion to their occurrence in the known map area. We used circuit theory to estimate landscape resistance to organism movement and gene flow, and compared the output across several scenarios: a habitat-quality map with artificial boundaries and no buffer, a map with a buffer composed of randomized habitat quality data, and a map with a buffer composed of the true habitat quality data. We tested the sensitivity of the randomized buffer to the possibility that the composition of the real but unknown buffer is biased toward high or low quality. We found that artificial boundaries result in an overestimate of landscape resistance.

**Conclusions/Significance:**

Artificial map boundaries overestimate resistance values. We recommend the use of a buffer composed of randomized habitat data as a solution to this problem. We found that resistance estimated using the randomized buffer did not differ from estimates using the real data, even when the composition of the real data was varied. Our results may be relevant to those interested in employing Circuitscape software in landscape connectivity and landscape genetics studies.

## Introduction

**Figure 1:**
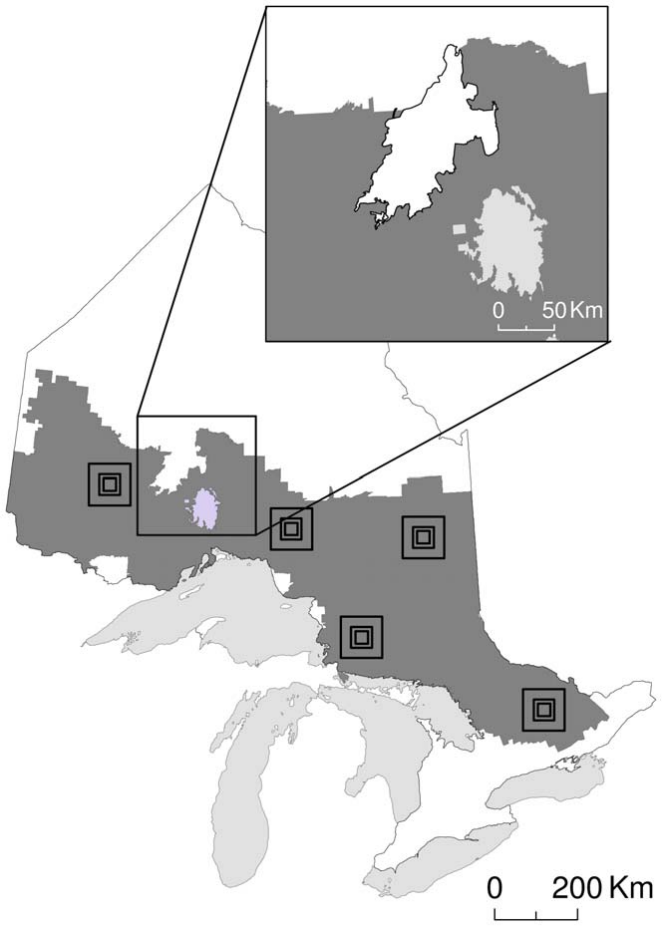
A map of Ontario, Canada. Dark grey depicts where detailed land-cover data exist in the province (forest resource inventory [FRI]). The boundaries of the dark grey area are “artificial” (except where bounded by water [light grey]), in that these boundaries exist because of a lack of data outside of the dark grey area; on the ground, habitat continues beyond these edges (white area). The inset map shows Wabakimi Provincial Park in Ontario (black outline), where we do not have FRI data but we know that the area contains suitable habitat. Models of gene flow across this region would attribute unrealistically high resistance to the habitat between the lake and the Park, while real organisms on the ground would be able to traverse the Park and surrounding habitat. Outlines of the 5 plots are shown.

Modeling habitat connectivity is integral to conservation planning [1], [2], as habitat corridors may act as conduits for organism movement and gene flow [e.g., 3–5]. The identification of functional corridors requires first mapping the permeability of the landscape to movement by the species of interest, and then modeling the organism's path of movement across the map. Recently developed connectivity models have moved beyond predicting single least cost paths to estimating multiple movement pathways [2], [6]–[9] and landscape resistance to movement [10], [11]. The nature of connectivity studies often requires that they occur at large spatial scales [e.g., 12,13]. As a result, many studies use remotely-sensed land-cover maps to represent the true landscape. Artificial boundaries of a map, boundaries that exist on the land-cover map but not on the ground, will arise in studies that span large areas where compatible remotely-sensed land-cover data might not exist for the entire region of interest. For example, in Ontario, Canada, detailed forest resource inventory (FRI) data exist only for commercially managed forests, even though forest exists beyond the inventoried area (Figure 1). Studies that span political jurisdictions may be particularly prone to having artificial boundaries, as comparable land-cover data may not be available on both sides of the political border. Likewise, researchers may be limited in map extent because of computational limitations of modeling software. We were interested in characterizing the effects of artificial map boundaries on calculations of landscape resistance to movement, and testing a possible solution for biased resistance values.

One promising modeling approach incorporates concepts from electrical circuit theory to model landscape permeability to both gene flow and individual movements [11], [14]. Rather than predicting specific, set movement pathways, circuit theory attempts to quantify the overall resistance of the landscape to organism movement. Resistance values that reflect the hypothesized ease of movement of individuals are assigned to raster map cells (nodes), creating a conductive surface. Exploiting the properties of random walks on electrical networks, effective landscape resistance can then be calculated between pairs of nodes based upon the commute times of random walkers; the expected length of a random walk to a node and back. Effective resistance on a landscape is a measure of isolation between cells on a raster grid. The result is a continuous surface of resistance estimates that incorporates all potential movement pathways into the measure of resistance. As this model is still in its infancy, we know little about how the results are affected by idiosyncrasies of the input data.

One of the reasons circuit theory is appealing as a model of gene flow and individual movement is that the incorporation of random walkers on networks to estimate landscape resistance allows resistance to decrease with increasing connectivity, path width, and path redundancy [11], [14]. This feature, however, leads to a potential problem; artificial boundaries may act as barriers to dispersal for the model organisms (random walkers) when, in reality, habitat beyond the boundary is available for use by real organisms. This could artificially decrease the number of paths connecting a landscape and increase perceived resistance and isolation of that landscape. This introduces a bias if researchers intend to compare the relative connectedness of the landscape between several sites. Those sites close to the map boundary may appear less connected than interior sites, when in fact this might be an artifact of the map boundary. If map extent was increased, random walkers would have more ‘room to run’, which would increase perceived connectivity between sites close to the map boundary. Several authors have alluded to the idea that artificial boundaries might influence the results of least cost path and landscape resistance models [2], [11], [15]–[18], but to our knowledge, no one has directly addressed the issue.

If model organisms (random walkers) of circuit theory are constrained by map boundaries, we predict that: 1) landscape resistance will be overestimated when the map has artificial boundaries within reach of random walkers; and 2) the addition of a buffer of habitat data around the artificial boundary will provide a more accurate resistance estimate. We created a buffer that was an extrapolation of the known habitat composition within the map (hereafter termed randomized buffer); habitat quality values were randomly assigned to map pixels in the buffer, in the same proportion as the map. We modeled landscape resistance to animal movement using circuit theory [11], [19], and compared the output from maps with artificial boundaries, maps with a randomized buffer, and maps with a buffer of true landscape data. We addressed the sensitivity of the randomized buffer by biasing the real buffer data toward both high and low habitat quality.

## Methods

We simulated a common scenario whereby a researcher wants to predict gene flow of a species between several sites of interest using a map representing the cost of movement for the species. Given the relatively large spatial scale of these types of studies, artificial boundaries might be produced due to the lack of available land-cover data (e.g., Figure 1). Some sites might be closer to the edge of the map than others. We used circuit theory [11], [14] to model the landscape's resistance to organism movement with the software CIRCUITSCAPE 3.5 [19]. We use the term resistance as a synonym of effective resistance and as an antonym of landscape connectivity. The term current is defined as the density of random walkers between two sites, and is negatively correlated with resistance (although it is not the inverse).

We demonstrated the effect of artificial boundaries on estimates of landscape resistance with an existing land-cover map (FRI data) of Ontario, Canada (Figure 1). We reclassified this map to represent the cost of movement by American martens (*Martes americana*), with a scale ranging from l (low) to 3 (high quality habitat). Habitat quality for martens was assessed from FRI using established habitat suitability models [e.g., 20]. The species of interest and area of study that we used for our simulation is irrelevant however, as we did not expect the outcome to be either species or landscape specific.

**Figure 2:**
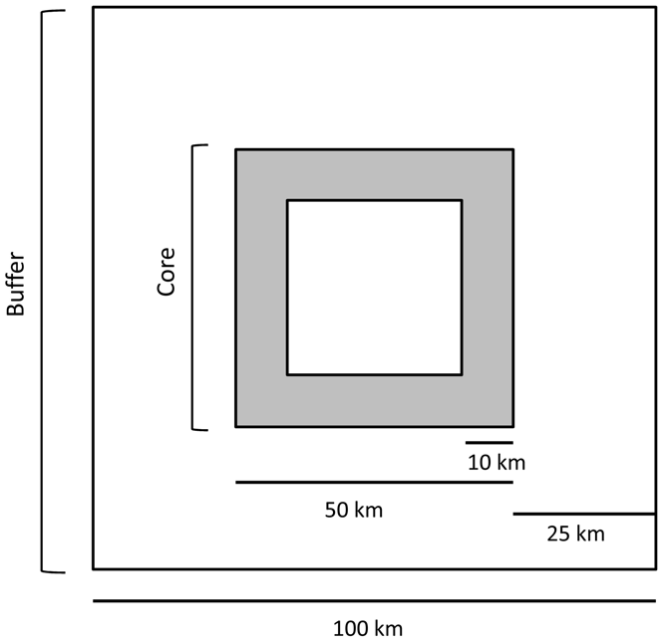
Dimensions of the plots. The core area includes both the outer core (grey) and the inner core (white).

Our study took place in 5 replicate 10,000-km^2^ plots (Figure 1). Our raster habitat map had a pixel size of 0.0625 km^2^ (*n* = 160,000 pixels per plot). Each plot consisted of a core area (2,500 km^2^) and a buffer (25-km wide) around the core (Figure 2). We divided the core area into an inner and outer core (Figure 2) because we were interested in comparing the effect of map boundaries on sites located close to the map boundary (outer core) to central sites (inner core). We estimated resistance between a source site randomly located within the core area (either inner or outer), and a destination site located 10 km from the source, in a random direction. We estimated landscape resistance between 10 randomly selected site pairs in each of the inner and outer cores, for each of the 5 plots (*n* = 100 pairs).

**Figure 3:**
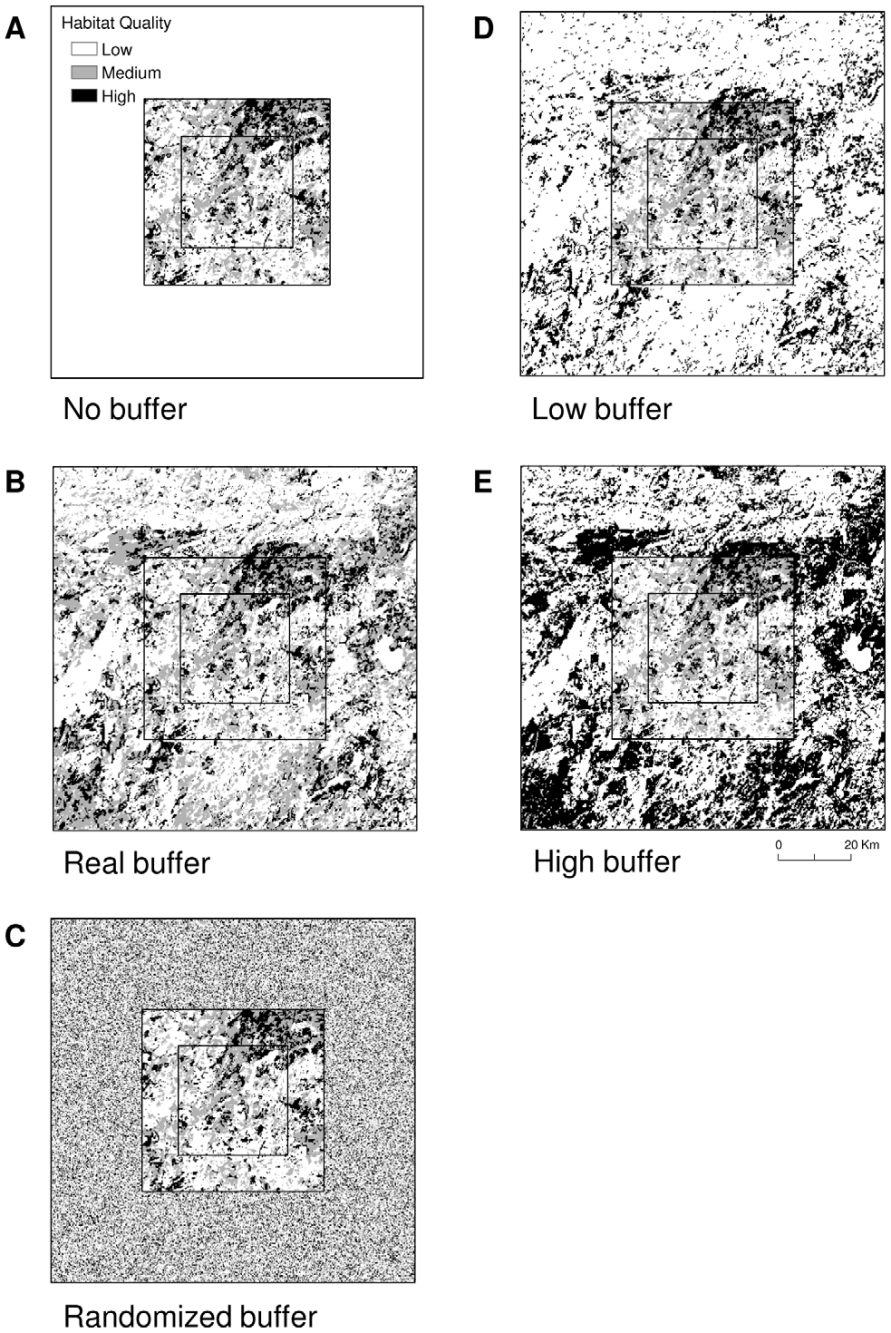
An example of one plot, showing the composition of suitable habitat for each scenario. a) a plot with habitat quality data in the core only (artificial boundary); b) a buffer around the core area composed of the true habitat quality data; c) a buffer composed of randomized habitat quality data; d) a buffer composed of true data that is biased toward low-quality; and e) a buffer composed of true data that is biased toward high-quality. Map pixels were 250-m by 250-m and all buffers were 25-km wide.

We assessed landscape resistance in multiple scenarios (Figure 3). The first scenario (no buffer) was a map with artificial boundaries (i.e., core area only), where we did not know the habitat composition on the ground beyond those boundaries (Figure 3a). The second scenario (real buffer) was a map of the core area plus a 25-km wide buffer composed of true landscape data as portrayed on the map (Figure 3b). It was meant to represent the true habitat on the ground beyond the map boundaries, and that which was unknown in the first scenario. The third scenario (randomized buffer) was a map of the core area plus a 25-km wide buffer composed of randomized habitat data (Figure 3c). This buffer was a substitute for the true data in scenario 2 (assuming the true data were unknown). We generated the randomized buffer by randomly assigning a habitat quality value between 1 and 3 to each grid cell in the buffer. The proportion of each value in the buffer was the same as the core area, as we wanted to emulate the situation where a researcher does not know the habitat composition beyond the boundaries of the map and the only land-cover information available is within the core area. Thus, the researcher could extrapolate beyond the map boundary.

Given the idiosyncrasies of the Ontario landscape, the proportion of each habitat quality value in the randomized buffer was similar to the proportion in the real buffer (Table 1). This ideal situation may not be generally applicable. Thus, we investigated the sensitivity of our randomized buffer to situations where the true landscape in the real buffer was less suitable (scenario 4, low buffer) or more suitable (scenario 5, high buffer) than the core. We generated these buffers by reclassifying the buffer from scenario 2 (real buffer) as follows: (1) we biased the buffer toward low quality by changing the pixels representing medium quality in the buffer to low quality (Figure 3d); and (2) we biased the buffer toward high quality by changing the medium quality pixels in the buffer to high quality (Figure 3e). We did not change the composition of the core area. The mean proportion of low-quality pixels across the 5 plots increased by 68% (SD = 73) between the real and low-biased buffers, whereas the mean proportion of high- quality pixels increased by an average of 206% (SD = 115) between the real and high-biased buffers (Table 1).

**Table 1:** The percentage of map pixels scored as low, medium, or high habitat quality^1^.

Plot	Scenario	Low	Medium	High
1	Real buffer	78	9	13
	Low quality	85	2	13
	Randomized buffer	79	7	14
	High quality	78	2	20
2	Real buffer	24	61	15
	Low quality	70	15	15
	Randomized buffer	25	60	15
	High quality	24	15	61
3	Real buffer	61	30	9
	Low quality	82	9	9
	Randomized buffer	59	35	6
	High quality	61	9	30
4	Real buffer	56	28	16
	Low quality	77	8	16
	Randomized buffer	51	31	18
	High quality	56	8	36
5	Real buffer	46	44	10
	Low quality	76	14	10
	Randomized buffer	35	56	9
	High quality	46	14	40

1 Values are a sum of the core area and the buffer, for each plot depicted in Figure 1.

We compared resistance, estimated using Circuitscape, between the five scenarios with Cohen's effect size (*d*) for paired comparisons (the difference between group means, divided by the pooled standard deviation; [21]). We pooled data over plots (*n* = 50). We interpreted effect size using Cohen's [21] general guidelines, whereby *d* = 0.2 is a small effect, *d* = 0.5 is a medium effect, and *d* = 0.8 is a large effect. Because our study is based on simulated data, we used effect size to assess differences between scenarios rather than test statistics; when using test statistics, a sufficiently large sample size will always suggest statistically significant differences.

## Results

**Figure 4:**
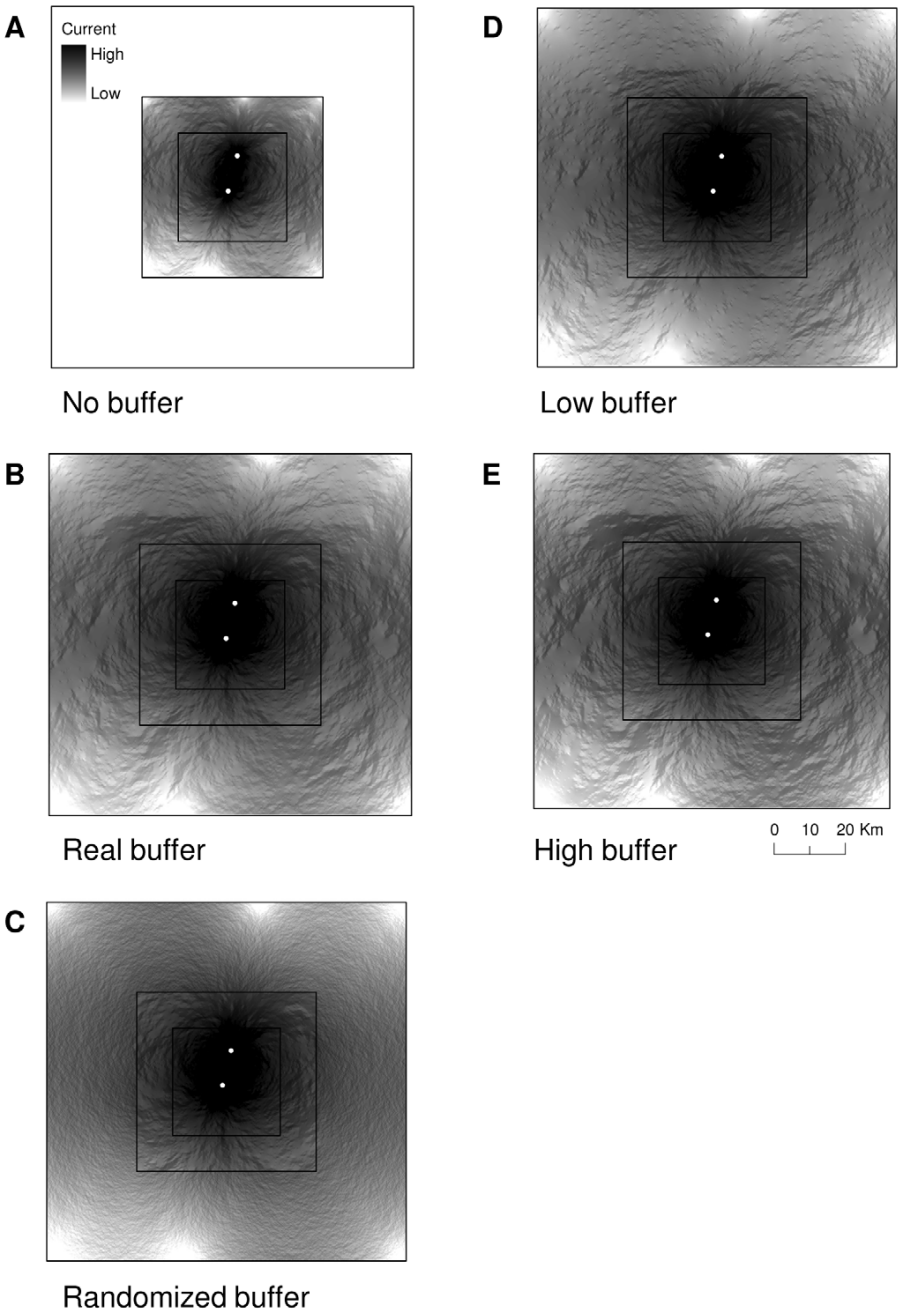
An example of the current between two randomly selected sites in the interior core. a) a plot with habitat data for the core only (artificial boundary); b) a buffer around the core area composed of the true habitat data; c) a buffer composed of randomized habitat data; d) a buffer composed of true data that is biased toward low quality; and e) a buffer composed of true data that is biased toward high quality. Estimates of current were generated using Circuitscape software [19] for the landscape depicted in Figure 3.

**Figure 5:**
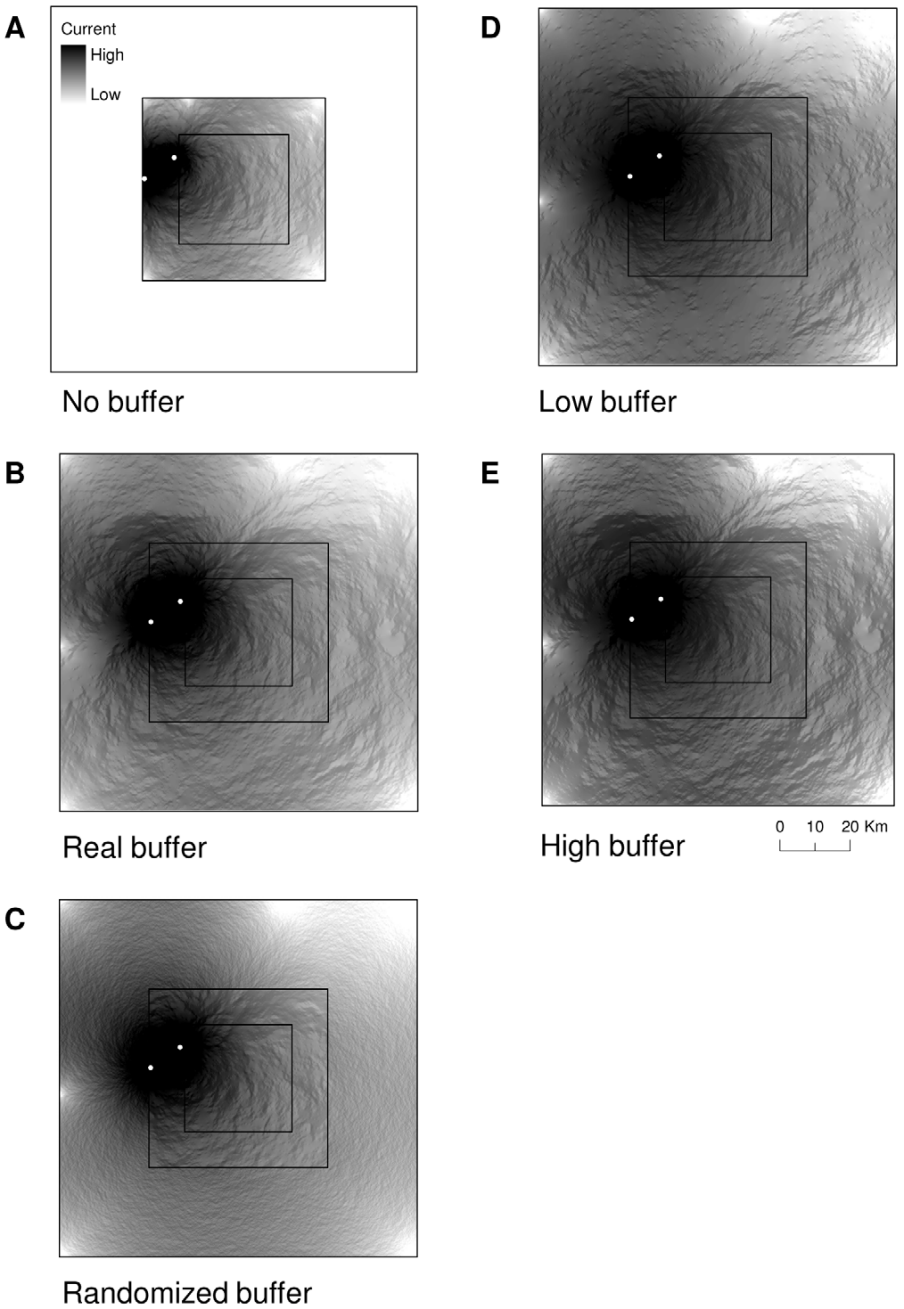
An example of the current between two randomly selected sites in the outer core. a) a plot with habitat data in the core only (artificial boundary); b) a buffer around the core area composed of the true habitat data; c) a buffer composed of randomized habitat data; d) a buffer composed of true data that is biased toward low quality; and e) a buffer composed of true data that is biased toward high quality. Estimates of current were generated using Circuitscape software [19] for the landscape depicted in Figure 3.

Resistance was overestimated in the scenario with no buffer relative to the buffer of real data (Figures 4–5) for sites in both the inner (*d* = 1.50) and outer (*d* = 0.90) cores, as indicated by large effect sizes (Table 2). When we used the randomized buffer however, resistance estimates were not different than estimates using a buffer of real data for sites in either the inner (*d* = 0.13) or outer (*d* = 0.002) cores, as indicated by small effect sizes (Table 2).

**Table 2:** The pair-wise mean difference in resistance between scenarios.^1^

Comparison^2^	Position	Mean Difference^3^	95% CI^4^	Effect Size^5^ (*d*)
No buffer vs. Real	Inner	8.84×10^−3^	8.28×10^−3^–9.40×10^−3^	1.50
	Outer	6.02×10^−2^	4.17×10^−2^–7.91×10^−2^	0.90
Randomized vs. Real	Inner	−2.24×10^−3^	−7.17×10^−3^–2.69×10^−3^	0.13
	Outer	−5.40×10^−5^	−6.24×10^−3^–6.13×10^−3^	0.002
No buffer vs. Low	Inner	7.18×10^−3^	4.53×10^−3^–9.83×10^−3^	0.75
	Outer	5.28×10^−2^	7.01×10^−2^–3.55×10^−2^	0.84
Randomized vs. Low	Inner	−3.90×10^−3^	−7.80×10^−3^– −3.93×10^−6^	0.28
	Outer	−7.46×10^−3^	−1.31×10^−2^– −1.84×10^−3^	0.37
No buffer vs. High	Inner	8.97×10^−3^	6.21×10^−3^–1.17×10^−2^	0.90
	Outer	6.45×10^−2^	4.45×10^−2^–8.44×10^−2^	0.90
Randomized vs. High	Inner	−2.11×10^−3^	−6.05×10^−3^–1.84×10^−3^	0.15
	Outer	4.23×10^−3^	−8.50×10^−4^–9.31×10^−3^	0.23

1 Across 10 random sites in each of 5 plots; *n* = 50.

2 The randomized buffer is composed of habitat data randomly assigned to pixels in proportion to the map itself, the real buffer is what is truly on the map, the low buffer is biased toward low-quality habitat data, and the high buffer is biased toward high quality data.

3 The difference is calculated as the first scenario minus the second scenario (e.g., for the first row, no buffer minus real buffer).

4 95% confidence interval of the mean pair-wise difference.

5 Cohen's effect size (*d*) for paired comparisons [21]; *d* = 0.2 is a small effect, d = 0.5 is a medium effect, *d* = 0.8 is a large effect.

Resistance was again overestimated for the scenario with no buffer relative to the buffer biased towards low quality for sites in both the inner (*d* = 0.75) and outer (*d* = 0.84) cores (Table 2). Conversely, when we used the randomized buffer, effect sizes were small; resistance did not differ from the buffer with low-biased data for sites in the inner (*d* = 0.28) or outer (*d* = 0.37) cores (Table 2).

We found that the scenario with no buffer overestimated resistance relative to when we biased the real buffer toward high habitat quality, for both the inner (*d* = 0.90) and outer (*d* = 0.90) cores (Table 2). We found no difference in resistance however, between scenarios with a buffer of randomized data and a buffer biased towards high quality, for sites in both the inner (*d* = 0.15) and outer (*d* = 0.23) cores, as indicated by small effect sizes (Table 2).

## Discussion

Artificial boundaries on maps biased landscape resistance estimated with circuit theory. As predicted, resistance was overestimated on maps with artificial boundaries, and the addition of a buffer removed the bias. This is because artificial boundaries limit the space available to random walkers, which artificially reduces the number of paths to each node, thus increasing perceived resistance. A buffer around the artificial boundary removed the barrier to movement and produced a more accurate estimate of resistance. Even if the true habitat composition in the buffer was different than the core (i.e., biased toward low or high quality), using a randomized buffer that was proportional to the composition of the core introduced less bias than not using a buffer. This was true for resistance estimates at sites close to the map boundary, as well as for centrally-located sites.

Our results are based on a relatively simple plot design; one with a direct path between two sites and no barriers or narrow habitat passages. We suspect that artificial boundaries will have a greater effect in more complex landscapes. For example, in Ontario the lack of FRI data for Wabakimi Provincial Park (Figure 1 inset) creates a narrow corridor of available habitat for which Circuitscape would attribute unrealistically high resistance. A buffer would allow model organisms to move through the Park and would, therefore, better model reality.

We recommend that a buffer be used when estimating landscape resistance in studies that would otherwise be influenced by map boundaries. This buffer need not be composed of map pixels randomly assigned to a habitat class, as in our simulation; the buffer could be based on satellite or other imagery if those data are available. Our map represented the landscape's permeability to movement by marten. The habitat suitability models used to build our map required information, such as the development stage and canopy closure of the forest, that were available only from FRI data. As such, the randomized buffer that we used was more appropriate than a buffer based on satellite imagery because we could not infer habitat suitability for martens otherwise. Users will need to consider which buffer is appropriate for their unique circumstance. The buffer width that will be sufficient to reduce bias in resistance estimates will also be project-specific. For many studies, habitat quality beyond the extent of the map is unknown, making arbitrary the choice of buffer width and composition. Computer memory limitations, resulting in the inability of Circuitscape to compute grids larger than six million cells [22], may ultimately restrict buffer width. Future work could build on our finding of no difference in resistance with biased buffers and address specifically the sensitivity of circuit theory to varying levels of landscape connectivity.

Circuit theory is an appealing model because, rather than modeling discrete paths that assume the disperser has prior knowledge of the landscape, it uses random walkers to model an overall resistance to movement. It is this theoretical strength, however, that causes the bias introduced by artificial boundaries on the map; map boundaries act as barriers to random walkers, thus inflating resistance estimates. We do not expect that artificial boundaries will affect the results of single-path least cost path models in the same way. As the use of multiple-path models, such as Circuitscape, becomes more widespread, users must address the bias that artificial boundaries on the map might introduce. We have presented a simple solution to the problem; a buffer that removes the barrier caused by map edges.
